# In this month’s Bulletin

**DOI:** 10.2471/BLT.24.000124

**Published:** 2024-01-01

In the editorial section, Jarbas Barbosa da Silva et al. (2) make the case for a digital transformation of primary health care.

Fid Thompson (5–6) reports on WHO’s efforts to improve the delivery of emergency care in humanitarian crises. Gamal Shiha talks to Gary Humphreys (7–8) about detection, prevention and treatment of hepatitis C in Egypt.

## Afghanistan, Albania, Argentina, Armenia, Belarus, Bolivia (Plurinational State of), Bosnia and Herzegovina, Burundi, Brazil, Cabo Verde, Central African Republic, Comoros, Colombia, Costa Rica, Cuba, Democratic Republic of the Congo, Djibouti, Ecuador, El Salvador, Ethiopia, Gabon, Guatemala, Guinea, Guinea-Bissau, Haiti, Honduras, Islamic Republic of Iran, Jamaica, Kenya, Kosovo, Kyrgyzstan, Lao People's Democratic Republic, Lebanon, Libya, Mali, Madagascar, Mauritania, Mozambique, Myanmar, Nepal, Nicaragua, Niger, Nigeria, Paraguay, Papua New Guinea, Peru, Philippines, Republic of Moldova, São Tomé and Príncipe, Somalia, South Sudan, Sri Lanka, Sudan, Syrian Arab Republic, Tajikistan, Thailand, Timor-Leste, Trinidad and Tobago, Tunisia, Ukraine, United Republic of Tanzania, Uzbekistan, Viet Nam, Yemen, Zimbabwe

### Vaccinating humanitarian workers 

Anne-Gaelle Selod et al. (46–57) describe the United Nations’ coordination of COVID-19 vaccine delivery.

## Bangladesh, Somalia 

### Achieving high levels of contraceptive use

Md Nuruzzaman Khan et al. (75–76) propose knowledge transfer between countries. 

## India

### Improving maternal and child nutrition services 

Christopher T Andersen et al. (9–21) correlate counselling provision with mothers’ knowledge and practices. 

## Global

### WHO essential medicines lists 

Enrico Costa et al. (22–31) track the increased number of orphan drugs listed over two decades.

### Tracking public opinion on COVID-19 vaccination

Xinyu Zhou et al. (32–45) correlate vaccine coverage with social media trends.

### Protecting public health in tobacco-growing countries

Raphael A Lencucha et al. (58–64) document industry influence.

### Preventing shigellosis 

William P Hausdorff et al. (65–74) summarize research on the value proposition of new vaccines.

### Sexual and reproductive health and rights

Tedros Adhanom Ghebreyesus et al. (74–75) point to a global health, gender equality and human rights imperative. 

